# Bioinformatics-based identification and validation of mitochondria-related genes associated with neonatal sepsis

**DOI:** 10.7717/peerj.20441

**Published:** 2025-12-17

**Authors:** Yu Zhong, Guilin Zhao, Shanshan Pu, Yushan Zhang, Dongju Pan, Xu Yang, Tingting Fu, Lu Chen, Chaofen Li, Xueyu Li, Zhi Li, Jun Wu, Shanping Chen, Zupeng Qiu, Ying Zhang, Fan Li

**Affiliations:** Department of Neonatology, Puer People’s Hospital, Pu’er, China

**Keywords:** Neonatal sepsis, Mitochondrial, Immune infiltration, Biomarkers

## Abstract

**Background:**

While mitochondrial involvement in sepsis has been widely studied, its role in neonatal sepsis (NESE) remains unclear. This study aimed to explore the molecular mechanisms of mitochondrial-related genes (MRGs) in NESE using bioinformatics analysis.

**Methods:**

This study utilized neonatal sepsis-related datasets GSE69686 and GSE95233. Differentially expressed genes (DEGs) were identified by comparing NESE and control groups. Subsequently, candidate genes were then selected by intersecting DEGs with MRGs. These candidate genes were further refined using least absolute shrinkage and selection operator (LASSO) regression and the Boruta algorithm to identify potential biomarkers. Expression levels and receiver operating characteristic (ROC) curve analyses of the candidate biomarkers were assessed in both datasets. To further investigate their mechanisms, functional enrichment, immune infiltration, and drug prediction analyses were conducted. Finally, biomarker expression was validated using reverse transcription-quantitative polymerase chain reaction (RT-qPCR).

**Results:**

A total of 24 candidate genes were obtained by overlapping 579 DEGs and 1,136 MRGs. *ACSL1*, * ALAS1*, *ALDH5A1*, *MTHFD2*, *PDSS1*, and *TSPO* were identified as biomarkers. The enrichment analysis revealed significant enrichment of *ACSL1*, *ALAS1*, *ALDH5A1*, * PDSS1*, and *TSPO* in the lysosome compartment. Moreover, there were significant differences in seven immune cells (M0 macrophage, activated NK cell, neutrophil, *etc*) between NESE samples and normal samples. Importantly, the correlation analysis revealed that the expression of *ACSL1* exhibited a negative correlation with CD8 + T cells, whereas it demonstrated a positive association with neutrophil. Additionally, it also found birch A and Tetrachlorodibenzo-p-dioxin simultaneously targeted *TSPO*, *MTHFD2*, *ALAS1*, *ALDH5A1*, *PDSS1*, and *ACSL1*. Importantly, the RT-qPCR results demonstrated that the expression results of *PDSS1*, *TSPO*, and *ALAS1* were consistent with the public database, showing significantly over-expressed in the NESE group.

**Conclusions:**

In this study, six mitochondria-related biomarkers in NESE were identified and preliminarily validated, which may provide novel insights into disease mechanisms and serve as a potential basis for future diagnostic and therapeutic exploration.

## Introduction

Neonatal sepsis (NESE) is a systemic inflammatory response syndrome in newborns caused by infections from various pathogens, including bacteria, viruses, fungi, and protozoa (Shane, Sánchez & Stoll, 2017). The disease often presents with initially mild symptoms but can progress rapidly, potentially leading to life-threatening conditions such as septic shock and multi-organ failure within a short period, thereby posing a serious threat to neonatal health (Procianoy & Silveira, 2020). Each year, approximately three million neonates globally are diagnosed with sepsis, with a mortality rate ranging from 11–19% (Fleischmann-Struzek et al., 2018). Moreover, survivors of NESE may also face long-term health issues and developmental deficits, such as cerebral palsy, intellectual disability, and developmental delays (Kavas et al., 2017). Therefore, early recognition of NESE is crucial for improving newborns survival and reducing long-term complications. However, NESE is often challenging to recognize due to its insidious clinical presentation in early stages, leading to delays in diagnosis and treatment. Currently, the clinical management of NESE typically involves the empirical use of broad-spectrum antibiotics, particularly in high-risk infants. While this strategy can sometimes improve the newborn’s prognosis, it carries risks such as adverse drug reactions, complications, and increased antibiotic resistance (Iroh Tam et al., 2023). Given the limitations of current diagnostic and therapeutic strategies, there is an urgent need to develop and validate reliable biomarkers to facilitate early diagnosis and enable more precisely targeted therapy for NESE.

Mitochondria are crucial cellular energy factories, primarily generating adenosine triphosphate through oxidative phosphorylation to supply cellular energy. Besides energy production, mitochondria participate in various physiological processes, including apoptosis, signal transduction, oxidative stress response, and regulation of cellular metabolism (Nunnari & Suomalainen, 2012). Dysfunctional mitochondria result in inadequate cellular energy supply and excessive reactive oxygen species (ROS) production, leading to oxidative stress. This stress damages intracellular biomolecules like DNA, proteins, and lipids, causing apoptosis and necrosis, thereby impairing tissue and organ function (Rong et al., 2021). Recent studies indicate that mitochondrial dysfunction plays a critical role in the pathogenesis of sepsis (Shu et al., 2024; Shu et al., 2023; Zou et al., 2022). In animal models of sepsis, cardiomyocyte mitochondria exhibit significant swelling, vacuole-like degeneration, and cristae rupture, accompanied by a marked decrease in mitochondrial membrane potential (Liang et al., 2018; Zhang et al., 2015). These alterations suggest that mitochondrial damage is linked to sepsis severity and poor prognosis. Although the connection between mitochondrial dysfunction and sepsis is established, its specific role and molecular mechanisms in NESE remain largely unknown. Due to its unique physiological characteristics, NESE may differ from adult sepsis in its pathological mechanisms. Therefore, exploring the specific role of mitochondrial metabolism in NESE is crucial for a comprehensive understanding of sepsis pathogenesis and for identifying new therapeutic strategies and interventions.

This study obtained datasets related to NESE from the Gene Expression Omnibus (GEO) database. Using a series of bioinformatics methods, including differentially expressed analysis, machine learning, expression evaluation and receiver operating characteristic (ROC) curves, the mitochondria-related biomarkers in NESE were screened. Based on biomarkers, the study further explored the relationship between these genes and immune infiltration and verified the expression of genes using reverse transcription-quantitative polymerase chain reaction (RT-qPCR), providing a diagnosis and treatment of NESE.

## Materials & Methods

### Data extraction

The GEO (http://www.ncbi.nlm.nih.gov/geo/) database offered the GSE69686 (platform: GPL13112) (https://www.ncbi.nlm.nih.gov/geo/query/acc.cgi?acc=GSE69686) and GSE95233 (platform: GPL570) datasets (https://www.ncbi.nlm.nih.gov/geo/query/acc.cgi?acc=GSE95233). As a training set, GSE69686 contained 64 NESE patient blood samples and 85 normal blood samples. The validation set GSE95233 consisted of 51 NESE patient blood samples and 22 control blood samples. Besides, 1,136 mitochondria-related genes (MRGs) were downloaded from the MitoCarta 3.0 (https://www.broadinstitute.org/mitocta) (Rath et al., 2021).

### Differential expression analysis

In the GSE69686 dataset, the limma package (v 3.50.1) (Ritchie et al., 2015) was utilized to analyze the differential expression between NESE and control samples. The *p* < 0.05 & —log_2_FoldChange(FC)— > 0.5 were used as a threshold to screen differentially expressed genes (DEGs). Volcano maps and heat maps were presented *via* ggplot2 package (v 3.3.2) (Gustavsson et al., 2022) and ComplexHeatmap package (v 2.14.0) (Gu, Eils & Schlesner, 2016) respectively for visualizing the expression of DEGs.

### Recognition and functional analysis of candidate genes

The intersection of screened DEGs and MRGs was determined as candidate genes. For investigating the role of candidate genes in biological functions and signaling pathways, Gene Ontology (GO) functions and Kyoto Encyclopedia of Genes and Genomes (KEGG) enrichment analyses of candidate genes were implemented by clusterProfiler package (v 3.14.3) (Yu et al., 2012) (*p*.adjust < 0.05). Specially, the GO terms included biological process (BP), cellular component (CC), and molecular function (MF). Furthermore, to investigate the protein-level interactions of the candidate genes, we utilized the STRING (http://string-db.org) database (Szklarczyk et al., 2021) and set the confidence scores parameter to ≥ 0.4, thereby constructing the protein-protein interaction (PPI) network.

### Screening for biomarkers

Further screening of candidate genes was performed by implementing least absolute shrinkage and selection operator (LASSO) regression analysis *via* the glmnet package (v 4.0-2) (Li, Lu & Yin, 2022a). Meanwhile, Boruta algorithm was applied to the further screening of candidate genes. The genes obtained by these two algorithms were intersected to obtain candidate biomarkers. Next, the expression of candidate biomarkers between NESE and control groups was evaluated in the both GSE69686 and GSE95233 datasets. Furthermore, the ROC curve was drawn by survivalROC package (v 1.0.3) (Heagerty, Lumley & Pepe, 2000) in two datasets to assess the diagnostic ability of candidate biomarker for NESE. Importantly, the candidate biomarkers with consistent expression trends, significant differences, and area under the curve (AUC) value greater than 0.7 in the two datasets were defined as biomarkers. Additionally, chromosomal locus mapping was drawn using Rcircos package (v 1.2.2) (Gu et al., 2014) to display the location of biomarkers on chromosomes.

### Functional enrichment analysis of biomarkers

The GeneMania database (http://www.genemania.org) (Franz et al., 2018) was used for in-depth exploration of other genes associated with biomarker function and their involvement in biological functions. Additionally, functional similarity between biomarkers was analyzed *via* the GOSemSim package (v 2.24.0) (Yu et al., 2010) (|cor| > 0.3, *p* < 0.05). To further understand the biological functions and pathways involved in the biomarkers, Spearman correlation analysis was performed between each biomarker and the remaining genes separately in the GSE69686 dataset using the psych package (v 2.2.9) (Robles-Jimenez et al., 2021) to obtain the correlation coefficients. The genes were then sorted according to the correlation coefficients. Using the sorting results, gene set enrichment analysis (GSEA) was implemented using the clusterProfiler package (v 3.14.3) (normalized enrichment score |NES| > 1, *p*.adjust < 0.05, q.value < 0.2). The background gene set was c2.cp.kegg.v7.4.symbols.gmt provided by GSEA (https://www.gsea-msigdb.org/) database.

### Immune infiltration analysis

The CIBERSORT algorithm was employed to evaluate the infiltration of 22 immune cell types between NESE and control groups based on all samples in the GSE69686 dataset. Samples with *p* >0.05 were excluded, and heat maps were generated to visually represent the proportion of immune cell infiltration. Then, immune cells expressed less than 30% in the total sample were excluded, and the remaining immune cells were included in the subsequent analysis. Meanwhile, the differences in infiltration of these immune cells between NESE and control groups were compared. Furthermore, Spearman correlation analysis was implemented to examine the associations among differential immune cells, as well as between differential immune cells and biomarkers (|cor| >0.3, *p* <0.05).

### Construction of molecular regulatory network and drug prediction

To further explore the regulatory mechanism of the biomarkers, the transcription factors (TFs), miRNAs and lncRNAs associated with the biomarkers were predicted. Firstly, the TF regulating biomarkers was predicted by NetworkAnalyst (https://www.networkanalyst.ca/) database (Zhou et al., 2019). Subsequently, the miRNAs targeting biomarkers were predicted using the DIANA microT database (https://bio.tools/DIANA-microT#!) (Tastsoglou et al., 2023) and TargetScan database (https://www.targetscan.org/vert_80/) (McGeary et al., 2019). Intersection of the miRNAs predicted from both databases was performed to identify key miRNAs. Following this, the lncRNAs with regulatory effects on these key miRNAs were predicted utilizing the miRNet database (https://www.mirnet.ca/miRNet/home.xhtml) (Chang et al., 2020). Based on these predicted regulatory factors, the TF-mRNA regulatory network and lncRNA-miRNA-mRNA network were constructed using Cytoscape software (v 3.8.2) (Shannon et al., 2003). On the other hand, to explore potential drugs to treat patients with NESE, potential drugs targeting biomarkers were predicted based on the Comparative Toxicogenomics Database (CTD) (http://ctdbase.org/) (Davis et al., 2023).

### Expression validation of biomarkers

Whole blood samples were collected from five NESE patients and five healthy children who visited Puer People’s Hospital between December 29, 2024, and February 28, 2025. These samples were used to validate the expression of candidate biomarkers *via* RT-qPCR. The study was approved by the Clinical Research Ethics Review Committee of Puer People’s Hospital (Ethics approval number: LUNSHEN 2024-25<Ke>-02). Written informed consent was obtained from the parents or legal guardians of all participants prior to sample collection. Total RNA was extracted from ten samples using TRIzol reagent (Ambion, Austin, TX, USA) in accordance with the manufacturer’s instructions. First-strand complementary DNA (cDNA) was synthesized from 1 µg of total RNA using the SureScript First-Strand cDNA Synthesis Kit (Servicebio, Wuhan, China). RT-qPCR was performed with the 2xUniversal Blue SYBR Green qPCR Master Mix (Servicebio, Wuhan, China). Primer sequences are provided in Table S1. GAPDH was used as the internal reference gene, and relative transcript levels were determined using the 2^−ΔΔCt^ method (Livak & Schmittgen, 2001).

### Statistical analysis

All statistical analyses were performed using R software (version 4.2.1; R Core Team, 2022). Correlations between the two groups were evaluated using Spearman’s rank correlation analysis. Group differences were assessed with the Wilcoxon rank-sum test. A two-tailed *p* value of less than 0.05 was considered to indicate statistical significance.

## Results

### A total of 24 candidate genes were significantly enriched in biosynthesis-related pathways

**Figure 1:**
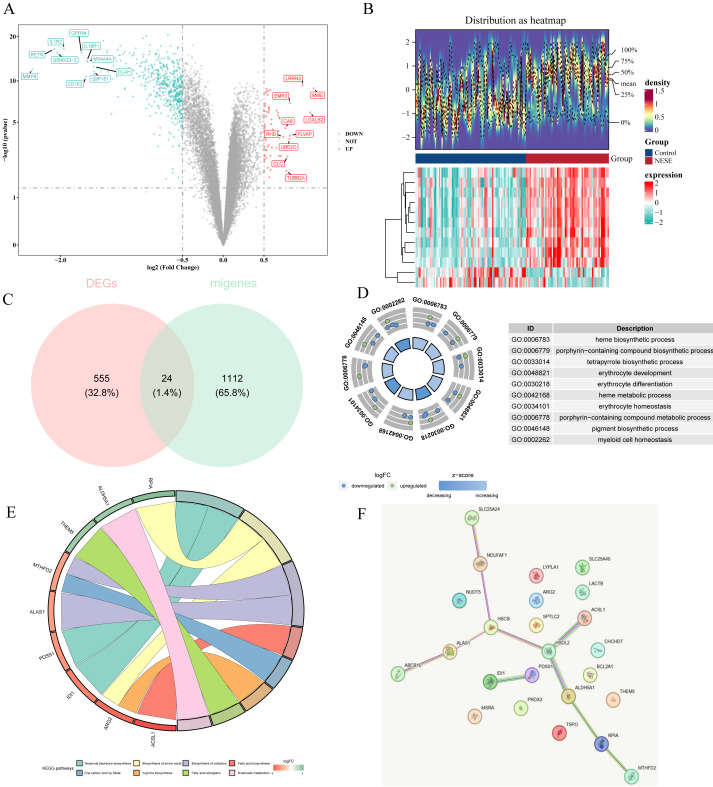
Identification of candidate genes and comprehensive analysis of their functional enrichment and protein-protein interactions. (A) The volcano plot of GSE69686. (B) The top 12 genes with the most remarkable expression changes of GSE69686. (C) Co expressed genes in NESE and MRG. (D) GO analysis. (E) KEGG pathway analysis. (F) PPI networks of candidate genes.

A total of 579 DEGs were identified between NESE and control groups, including 65 up-regulated genes and 514 down-regulated genes (Figs. 1A–1B). Subsequently, 24 candidate genes were obtained by overlapping 579 DEGs and 1,136 MRGs (Fig. 1C). Based on *p*.adjust < 0.05, 45 GO terms were enriched by these candidate genes, which contained 30 BPs, five MFs, and five CCs. Specially, these GO terms included heme biosynthetic process, porphyrin-containing compound biosynthetic process, myeloid cell homeostasis, *etc* (Fig. 1D). Additionally, eight KEGG pathways were found to be associated with these candidate genes, such as fatty acid biosynthesis, biosynthesis of amino acids, arginine biosynthesis and on on (Fig. 1E). These results suggested that the candidate genes might affect the occurrence of NESE by participating in these biosynthesis processes. Furthermore, a PPI network was constructed according to these candidate genes. Among them, *HSDL2* exhibited a strong interaction with *ACSL1* and *ALDH5A1* (Fig. 1F).

### ACSL1, ALAS1, ALDH5A1, MTHFD2, PDSS1 and TSPO were identified as biomarkers

**Figure 2:**
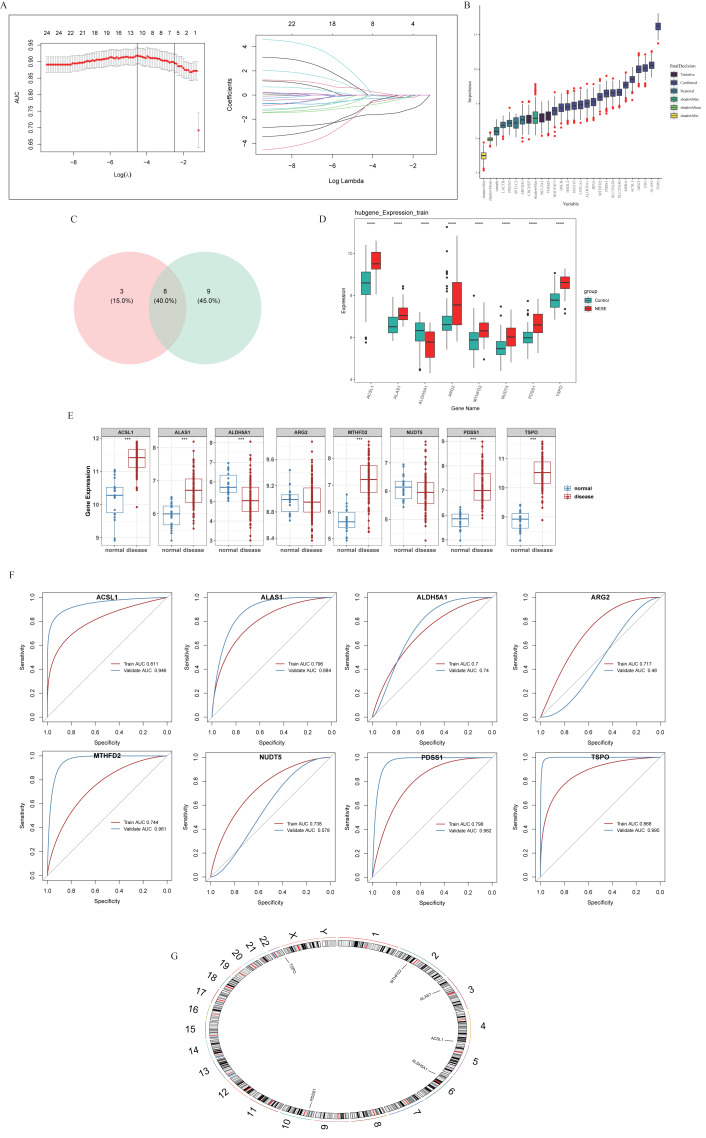
Identification of biomarkers through machine learning methods. (A) LASSO regression analysis. (B) The Boruta method. (C) Venn diagram showed that eight potential markers were found by LASSO and Boruta. (D) Core genes expression in the training set. (E) Core genes were expressed in validation sets. (F) ROC curves of core genes in training sets and validation sets. (G) Chromosomal localization of core genes.

A total of 11 and 17 genes were identified by LASSO regression analysis (Fig. 2A) and Boruta algorithm (Fig. 2B), respectively. Then, eight candidate biomarkers, namely *TSPO, PDSS1, ALAS1, ACSL1, MTHFD2, NUDT5, ALDH5A1*, and *ARG2* were gained through taking the intersection of these two parts of genes (Fig. 2C). Expression assessments performed at GSE69686 and GSE95233 datasets revealed that the expression of *ACSL1, ALAS1, ALDH5A1, MTHFD2, PDSS1,* and *TSPO* trended consistently across the two datasets and differed significantly in the NESE and control groups. Specifically, with the exception of *ALDH5A1*, the remaining candidate biomarkers were significantly more highly expressed in the NESE group (Figs. 2D–2E). Moreover, the AUC values of these six candidate biomarkers all exceeded 0.7 (Fig. 2F). In summary, *ACSL1, ALAS1, ALDH5A1, MTHFD2, PDSS1,* and *TSPO* were identified as biomarkers. Additionally, chromosome localization results indicated that *ACSL1, TSPO, ALAS1, ALDH5A1, MTHFD2*, and *PDSS1* were located on chromosomes 4, 22, 3, 6, 2, and 10, respectively (Fig. 2G).

### Biomarkers were mostly enriched on the Lysosome

**Figure 3:**
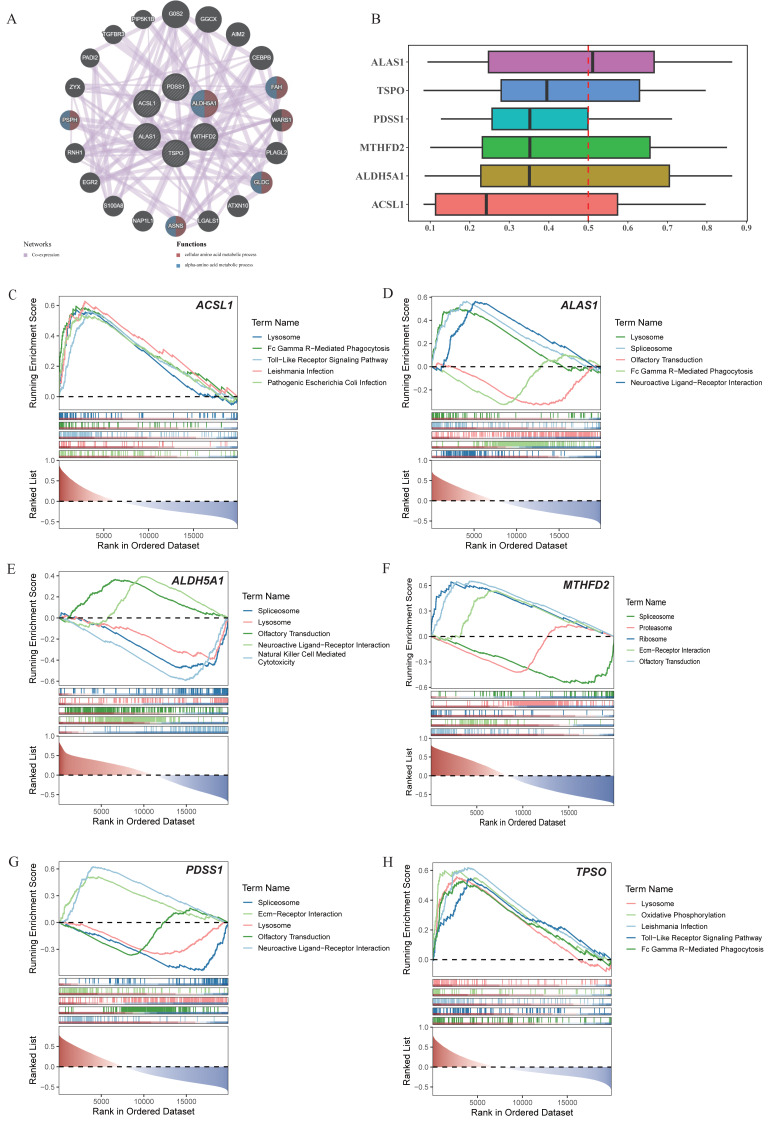
Co-expression networks, functional similarities, and pathway enrichments of core genes. (A) Co-expression network of core genes and functional genes. (B) Functional similarity analysis among six core genes. (C-H) GSEA enrichment analysis of *ACSL1*, *ALAS1*, *ALDH5A1*, *MTHFD2*, *PDSS1* and *TSPO*.

Through the GeneMania database, we explored the biomarker functions associated with the other genes and their involved biological functions. The biomarkers were found to be associated with 20 genes, including *FAH, WARS1, GLDC, ASNA, PSPH* and others. These genes collectively participated in the cellular amino acid metabolic process as well as the alpha-amino acid metabolic process (Fig. 3A). Meanwhile, *PDSS1, MTHFD2*, and *ALDH5A1* were functionally relatively similar (Fig. 3B). The enrichment analysis revealed significant enrichment of *ACSL1, ALAS1, ALDH5A1, PDSS1*, and *TSPO* in the lysosome compartment. Additionally, *ACSL1, ALAS1*, and *TSPO* were significantly enriched in Fc gamma r-mediated phagocytosis pathway. Furthermore, *ACSL1* and *TSPO* exhibited significant enrichment in toll-like receptor signaling pathway. Lastly, neuroactive ligand–receptor interaction pathway showed significant enrichment of *ALAS1, ALDH5A*, and *PDSS* (Figs. 3C–3H).

### Biomarkers were closely related to seven different immune cells

**Figure 4:**
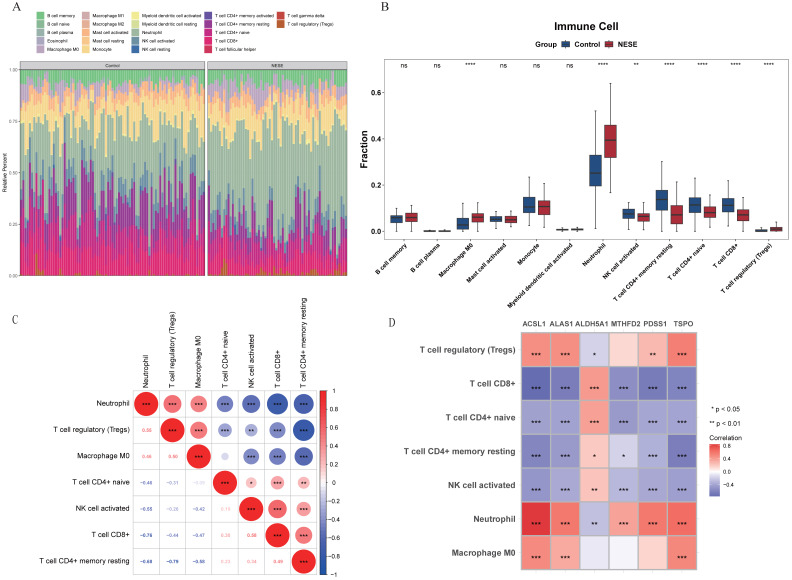
Immune infiltration of NESE. (A) Infiltration abundance heat maps of immune cells in NESE and normal samples. (B) Differences in the abundance of immune cell infiltration between NESE and normal groups. (C) The correlation between differentially expressed immune cells. The color of the circles represents the correlation coefficient (red for positive, blue for negative). The size of the circles indicates the strength of the correlation. The numbers in the lower triangle represent the specific Pearson correlation coefficients. Asterisks denote statistical significance: ^∗^*P* < 0.05, ^∗∗^*P* < 0.01, and ^∗∗∗^*P* < 0.001. (D) The correlation between core genes and differentially expressed immune cells. The color of the squares represents the correlation coefficient (red for positive, blue/purple for negative). The color intensity indicates the strength of the correlation. The *x*-axis represents the hub genes and the *y*-axis represents the immune cells. Asterisks denote statistical significance: ^∗^*P* < 0.05, ^∗∗^*P* < 0.01, and ^∗∗∗^*P* < 0.001.

In the GSE69686 dataset, the infiltration proportion of 22 immune cells in NESE and control groups were displayed through the heat map (Fig. 4A). After eliminating immune cells with expression levels lower than 30% in the total sample, 12 immune cells were included in the subsequent analysis. There were significant differences in seven immune cells between NESE samples and control samples, which were M0 macrophage, activated NK cell, neutrophil, resting memory CD4^+^ T cell, naive CD4^+^ T cell, CD8^+^ T cell, regulatory T cell (Tregs). Among them, M0 macrophage, neutrophil, Treg were significantly more infiltrated in the NESE group, while other immune cells were the opposite (Fig. 4B). Furthermore, the seven differential immune cells exhibited a robust correlation, characterized by a strong negative association between neutrophil and CD8^+^ T cell, as well as a pronounced positive relationship between neutrophil and Tregs (Fig. 4C). Additionally, the correlation analysis between differential immune cells and biomarkers revealed a significant negative association between *ACSL1* and CD8^+^ T cell (cor = −0.76), while the *ACSL1* exhibited a significant positive correlation with neutrophil (cor = 0.85) (Fig. 4D, Table S2).

### Biomarkers were regulated by multiple factors simultaneously

**Figure 5:**
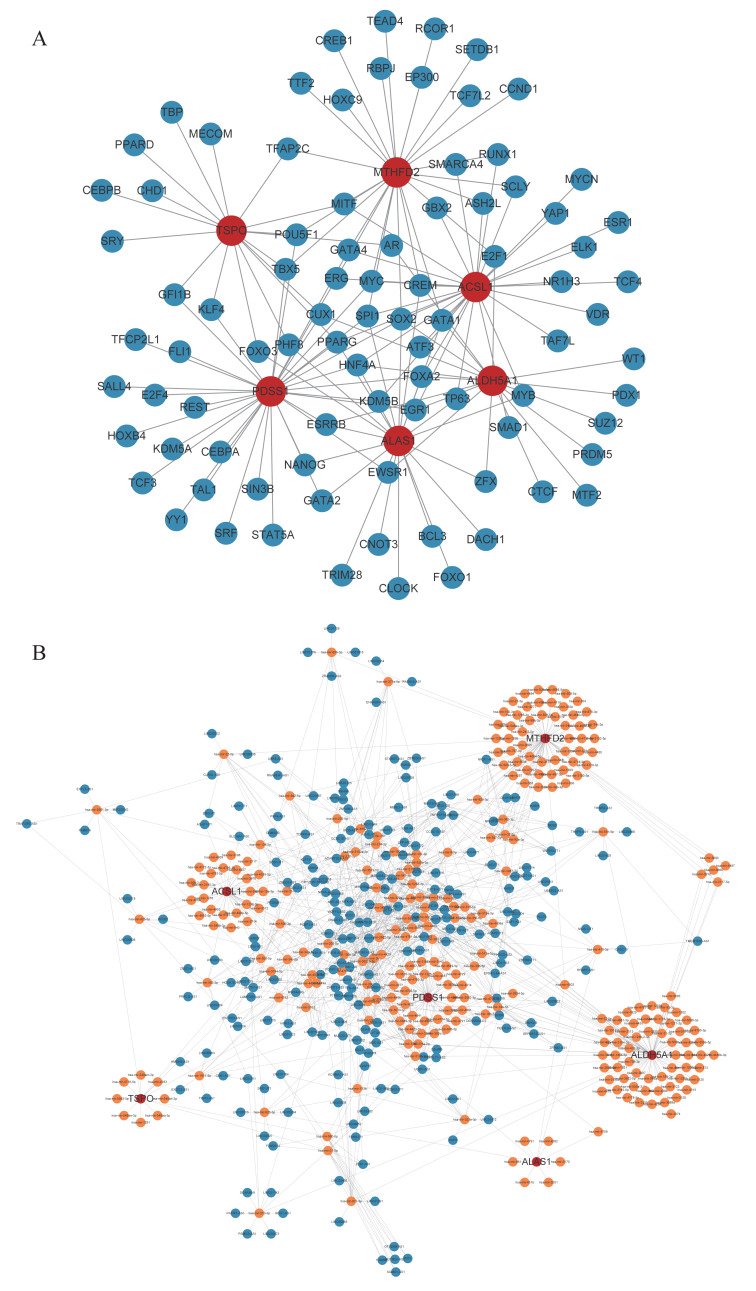
Competing endogenous RNAs network. (A) TF-core gene regulatory network. (B) lncRNA-miRNA-mRNA regulatory network.

Through prediction, we found that there were 88 TFs targeting biomarkers. The TF-mRNA regulatory network containing 93 nodes and 143 edges was constructed. Among them, MITF simultaneously targeted *TSPO, MTHFD2*, and *ACSL1. SOX2* also targeted *MTHFD2, ACSL1, ALDH5A1, ALAS1, PDSS1* (Fig. 5A). Meanwhile, a lncRNA-miRNA-mRNA network was constructed based on biomarkers, miRNAs, and top10 lncRNAs regulated miRNAs (Fig. 5B). Specially, *TNK2-AS1* regulated *PDSS1* and *PDSS2* through hsa-mir-4761-3p and hsa-mir-1278, respectively (Table S3).

### A variety of drugs had a potential relationship with biomarkers

**Figure 6:**
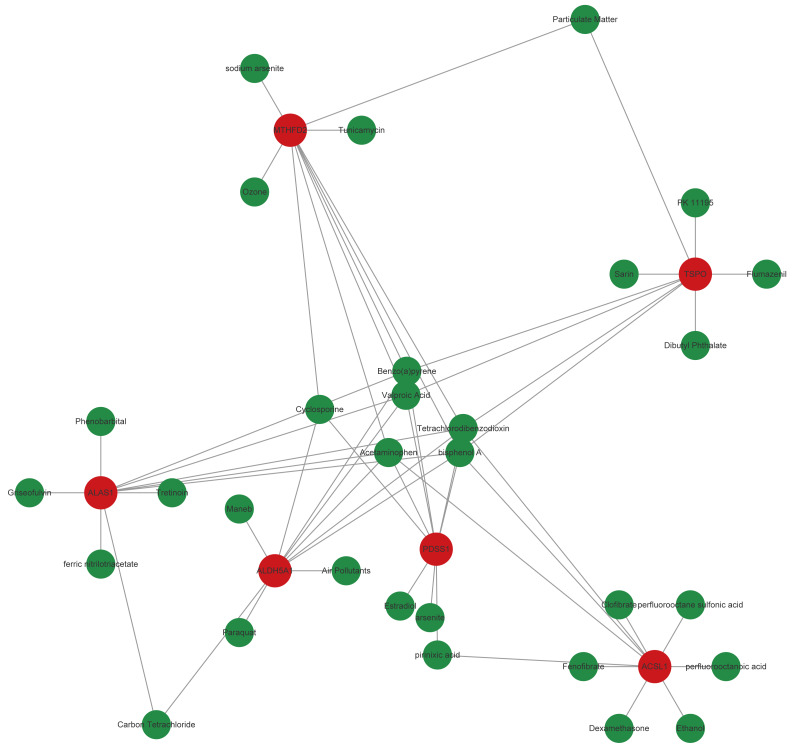
Core genes-potential drug interaction network.

Based on the CTD database, potential drugs targeting biomarkers were searched. Subsequently, the top10 drugs predicted by each mRNA were selected, and a gene-drug network containing 541 nodes and 957 edges was constructed (Fig. 6, Table S4). Specially, Birch A and Tetrachlorodibenzo-p-dioxin simultaneously targeted *TSPO, MTHFD2, ALAS1, ALDH5A1, PDSS1*, and *ACSL1*.

### The expression verification results of biomarkers

**Figure 7:**
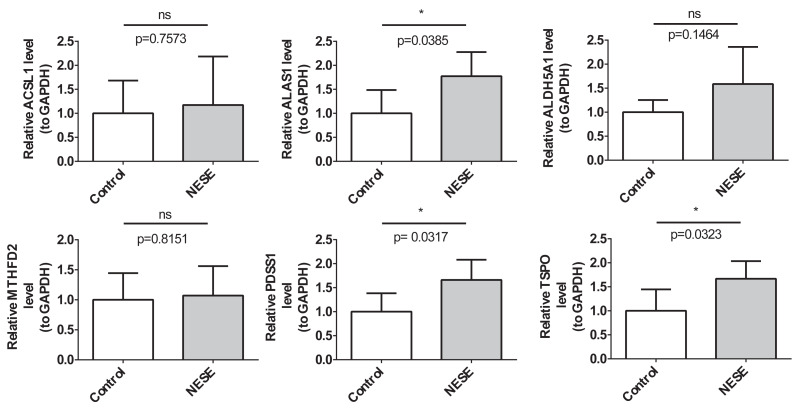
The expression verification results of NESE biomarkers.

The RT-qPCR results demonstrated that the expression results of *PDSS1, TSPO*, and *ALAS1* were consistent with the public database, showing significant up-regulation in the NESE group. Additionally, the expression trends of *MTHFD2* and *ACSL1* were also consistent with the public database; however, no statistical difference was observed between the NESE group and the control group (Fig. 7).

## Discussion

NESE is a prevalent infectious disease in newborns and has become the leading cause of neonatal mortality globally (Shane, Sánchez & Stoll, 2017). Mitochondria plays a crucial role in cellular energy metabolism and regulate immune, inflammatory, and metabolic processes. The dysfunction is potentially pivotal in the pathogenesis of NESE (Plotnikov et al., 2019). However, the specific role of mitochondria in NESE remains unclear. Therefore, elucidating the role of MRGs in NESE is essential for improving its diagnosis and treatment.

In this study, the dataset of NESE were accessed for differential gene analysis and a set of MRGs linked to NESE were identified. Subsequently, two machine learning algorithms were used to screen the candidate biomarkers. ROC curves were then plotted to evaluate their diagnostic value. This process led to the identification of six biomarkers in NESE (*ACSL1, ALAS1, ALDH5A1, MTHFD2, PDSS1, and TSPO*).

*ACSL1* is an enzyme crucial for lipid metabolism, responsible for converting free long-chain fatty acids into acyl-CoAs. These acyl-CoAs then enter the mitochondria for β-oxidation, producing Acetyl-CoA, which supplies energy to the cell (Li et al., 2022b). Multiple researches have confirmed an increased transcript abundance of *ACSL1* during sepsis (Khaenam et al., 2014; Pankla et al., 2009; Parnell et al., 2013). This increase may be due to the fact that high *ACSL1* expression enhances fatty acid activation and peroxidation, producing harmful lipid peroxides that damage mitochondrial membranes and lead to mitochondrial dysfunction. Mitochondrial dysfunction triggers oxidative stress and apoptosis, which in turn activate the inflammatory response and exacerbate sepsis. Further studies have shown that *ACSL1* plays a key role in inflammasome activation and pro-inflammatory cytokine release in macrophages, with its high expression closely linked to these processes, and the knockdown of *ACSL1* reduced NLRP3 inflammasome activation, IL-1β release, and lysosomal damage (Kalugotla et al., 2019). These findings are consistent with the higher expression of *ACSL1* in NESE obtained in this study, suggesting that *ACSL1* may play a pathological role in NESE by regulating mitochondria.

*ALAS1* is a mitochondrial enzyme crucial for heme synthesis (Lian et al., 2018). Heme is a core component of hemoglobin and is essential for cellular respiration, oxygen transport, mitochondrial function, and redox homeostasis. Studies have shown that elevated hemoglobin levels can cause oxidative damage and increase ROS, disrupting mitochondria and leading to mitochondrial DNA damage (Liang & Godley, 2003; Ucar et al., 2021). Our results revealed that *ALAS1* expression is up-regulated in NESE patients. *ALAS1* may promote heme biosynthesis, inducing oxidative stress that leads to mitochondrial DNA damage and contributes to the pathology of NESE.

*MTHFD2*, a mitochondria-associated one-carbon metabolizing enzyme, is crucial for regulating cell proliferation, signal transduction, and immune responses. It has been shown that *MTHFD2* maintains the integrity of the mitochondrial respiratory chain and prevents mitochondrial dysfunction (Yue et al., 2020). Our study found that *MTHFD2* was significantly overexpressed in neonates with sepsis, suggesting it may play a protective role in the pathological process of NESE. However, current research indicates a complex role for *MTHFD2* in inflammation and immunity. On one hand, *MTHFD2* regulates purine synthesis and signaling in activated T cells, promoting the proliferation of Th17 cells and the production of inflammatory cytokines. This contributes to the inflammatory response, leading to tissue damage and multi-organ failure (Ducker et al., 2016; Sugiura et al., 2022). On the other hand, *MTHFD2*’s regulatory effect on macrophage and T-cell polarization may trigger immune dysregulation in sepsis, affecting patients’ immune response capacity (Shang et al., 2023). Consequently, the specific mechanism of *MTHFD2* in NESE requires further study and validation.

*PDSS1* is a crucial mitochondrial enzyme involved in the synthesis of coenzyme Q10. Coenzyme Q10 is a vital component of the electron transport chain and is essential for cellular energy metabolism and antioxidant processes (Zhao et al., 2022). Our study found that *PDSS1* is significantly over-expressed in neonates with sepsis. Therefore, we hypothesize that *PDSS1* may help alleviate oxidative stress and cellular damage in NESE by promoting coenzyme Q10 synthesis and enhancing cellular antioxidant capacity. However, our findings differ from some literature reports indicating CoQ10 deficiency in sepsis patients (Coppadoro et al., 2013; Donnino et al., 2015). This discrepancy may result from various factors, including post-translational regulation, other constraints in the metabolic pathway, or sepsis-induced metabolic reprogramming, which may affect CoQ10 biosynthesis or utilization. Additionally, sepsis-induced oxidative stress and inflammation may disrupt CoQ10 synthesis or accelerate its consumption (Donnino et al., 2011). Given these contradictory results, further research is needed to understand the relationship between high *PDSS1* expression and CoQ10 synthesis, as well as the precise role and prognostic significance of *PDSS1* in NESE.

*TSPO* is a mitochondrial outer membrane protein that influences cell survival and function by regulating mitochondrial function, cellular stress response, and apoptosis (Papadopoulos et al., 2006). A study suggests that in sepsis-associated liver injury, *TSPO* may influence the pathological process by promoting inflammatory responses and exacerbating liver injury. Specifically, knockdown of *TSPO* in a mouse model of sepsis inhibited the polarization of hepatic macrophages towards a pro-inflammatory M1 phenotype and promoted their polarization towards an anti-inflammatory M2 phenotype, leading to a reduction in serum levels of pro-inflammatory cytokines (Jin et al., 2022). This suggests that *TSPO* may play a key role in regulating inflammatory responses and immune cell function in sepsis. Our findings indicate that *TSPO* expression is significantly upregulated in sepsis patients, supporting its potential role in promoting inflammatory and immune responses.

*ALDH5A1* encodes succinate semialdehyde dehydrogenase, an enzyme involved in mitochondrial glutamate metabolism, which enables mitochondrial energy metabolism and contributes to the maintenance of mitochondrial integrity. Reduced *ALDH5A1* is suggested to lead to mitochondrial dysfunction, affecting oxidative phosphorylation. This dysfunction is characterized by reduced ATP production, increased oxidative stress, and impaired mitochondrial dynamics, ultimately causing apoptosis and inflammatory responses (Brown et al., 2020). In NESE, a reduction in *ALDH5A1* may exacerbate mitochondrial dysfunction and oxidative stress, thereby promoting disease progression.

To further elucidate the role of biomarkers in NESE, the study conducted GSEA enrichment analysis. The analysis showed that these biomarkers were mainly enriched in the lysosomal pathway, Fc γ R-mediated phagocytosis, and Toll-like receptor (TLR) signalling pathway. Firstly, lysosomes are crucial for maintaining cellular homeostasis by removing intracellular waste products, damaged structures, and damaged mitochondria during autophagy, which is particularly important in NESE-induced cellular stress and injury (Kuk et al., 2023). Secondly, Fc γ R-mediated phagocytosis is vital in the immune response, controlling infection by recognizing and removing antibody-labeled pathogens and regulating immune cell activation and function (Bournazos & Ravetch, 2015). Finally, studies have shown that altered function of toll-like receptors (TLRs) may increase susceptibility to infection and contribute to persistent inflammation in both full-term and preterm newborns (Dias et al., 2021; Glaser & Speer, 2013). Also, over-activation of TLR signalling may lead to inflammatory storms and tissue damage (Kumar, 2020). Taken together, these biomarkers may play a central role in the development and progression of NESE by regulating cell homeostasis, immune defense, and inflammation.

This study found significantly higher infiltration of M0 macrophages, neutrophils, and regulatory T cells, and relatively lower infiltration of activated NK cells, resting memory CD4^+^ T cells, naïve CD4^+^ T cells, and CD8^+^ T cells in neonates with sepsis. Notably, a strong negative correlation was observed between neutrophils and CD8^+^ T cells, while a significant positive correlation was found between neutrophils and Tregs. Neutrophils are key components of the innate immune system, responding rapidly in the early stages of sepsis by increasing in number, migrating to infection sites, and exerting phagocytic and bactericidal effects (Hazeldine, Hampson & Lord, 2014). However, overactivation of neutrophils can release large amounts of inflammatory mediators, inducing endothelial cells to express pro-inflammatory molecules, which exacerbates the inflammatory response and tissue damage (Zhang et al., 2023). In NESE, levels of Tregs are significantly elevated and may correlate with disease severity and mortality. Tregs are crucial in maintaining immune homeostasis by suppressing excessive immune responses. Experimental studies have shown that simvastatin treatment or Rho kinase activity inhibition can reduce Treg levels in sepsis models, potentially helping to restore immune homeostasis and improve sepsis outcomes (Hasan et al., 2013; Zhang et al., 2012). In addition, CD8^+^ T cells are vital in combating infections by recognizing and killing pathogen-infected cells. In sepsis patients, a reduction in the number of CD8^+^ T cells may lead to compromised immune function, potentially resulting in an increased susceptibility to infections and a diminished capacity to clear pathogens (Berton et al., 2023; Guo et al., 2021). Programmed death molecule 1 (PD-1) is an immune checkpoint molecule that regulates the immune response by binding to CD8^+^ T cells and inhibiting their activity. In sepsis, upregulation of PD-1 may impair CD8^+^ T cell function and exacerbate disease progression (Akhmaltdinova et al., 2022). Further studies revealed a significant negative correlation between *ACSL1* and CD8^+^ T cells, and a significant positive correlation between *ACSL1* and neutrophils. These results suggest that *ACSL1* may influence the activation of both the innate and acquired immune systems in sepsis by regulating the function of different immune cells.

In this study, six mitochondria-related biomarkers in NESE were identified through bioinformatics, which may provide valuable insights for early diagnosis and the development of effective therapeutic strategies. However, several limitations should be acknowledged. First, this research was based on publicly available transcriptomic data, and although preliminary validation was performed through immune infiltration analysis and RT-qPCR, the sample size was relatively small. Additional validation using larger, multi-center clinical cohorts is required to confirm the robustness and generalizability of our findings. Second, the precise molecular mechanisms by which these mitochondria-related biomarkers contribute to the pathogenesis and prognosis of NESE remain to be fully elucidated. Notably, some genes may play dual roles in either promoting disease progression or participating in organismal protection, depending on the immune environment and pathological conditions. Future research is warranted to elucidate the regulatory mechanisms governing the expression of these genes, dissect their interconnections within functional networks, and evaluate their contributions to disease pathogenesis.

## Conclusions

In summary, six mitochondria-associated biomarkers in NESE were identified through bioinformatics analysis and machine learning methods. Subsequent immune infiltration analysis and RT-qPCR validation supported their potential involvement in the disease process. These findings enhance our understanding of the molecular mechanisms underlying NESE and lay the groundwork for future exploration of their potential utility in diagnosis and targeted treatment of neonatal sepsis.

##  Supplemental Information

10.7717/peerj.20441/supp-1 Supplemental Information 1 MIQE checklist

10.7717/peerj.20441/supp-2 Supplemental Information 2 Gene primer sequence table

10.7717/peerj.20441/supp-3 Supplemental Information 3 The correlation analysis between differential immune cells and biomarkers

10.7717/peerj.20441/supp-4 Supplemental Information 4 mRNA–miRNA Interaction Table

10.7717/peerj.20441/supp-5 Supplemental Information 5 Drug–mRNA Interaction Table

10.7717/peerj.20441/supp-6 Supplemental Information 6 Data

10.7717/peerj.20441/supp-7 Supplemental Information 7 PCR Experiment Report

10.7717/peerj.20441/supp-8 Supplemental Information 8 English translations for the Chinese text in the QPCR Experiment Report

## Additional Information and Declarations

### Data Availability

The following information was supplied regarding data availability: The data is available in the Supplemental Files. The raw sequence data are available at NCBI GEO: GSE69686 and GSE95233.
