# Supplementary material for: Bioinformatics-based identification and validation of mitochondria-related genes associated with neonatal sepsis
Source: PeerJ. 2025 Dec 17;13:e20441. doi: 10.7717/peerj.20441 (PMC12717851; doi:10.7717/peerj.20441)
Supplement: Supplemental Information 2 [file peerj-13-20441-s002.docx]

Supplementary Table 1. Gene primer sequence table

| **primer** | **sequence** | |
| --- | --- | --- |
| MTHFD2 F | GAACTGGCATTCCAACCCTA | |
| MTHFD2 R | CCCATCTGTGTGCAGTAACA | |
| PDSS1 F | GACCAATTATTGTGGCGCTAATG |  |
| PDSS1 R | AGACTAGCAGTGTGGATCATTTC |  |
| TSPO F | CCTACCTGGTCTGGAAAGAG |  |
| TSPO R | TCGGGCACCAAAGAAGATG |  |
| ALDH5A1 F | TGTACTGTCGTGGTGAAGCC |  |
| ALDH5A1 R | ACACCTGAAGGAATCCCAGC |  |
| ALAS1 F | GGTTGTGTTGGAGGGTACAT |  |
| ALAS1 R | CAGAGAGGTGGTGAAGATGAAG |  |
| ACSL1 F | AAAGACAGATGGGAGGAGACC |  |
| ACSL1 R | GTTGGTCGGAAGAGTACGCA |  |
| GAPDH F | CGAAGGTGGAGTCAACGGATTT |  |
| GAPDH R | ATGGGTGGAATCATATTGGAAC |  |
