# Supplementary material for: Bioinformatics-based identification and validation of mitochondria-related genes associated with neonatal sepsis
Source: PeerJ. 2025 Dec 17;13:e20441. doi: 10.7717/peerj.20441 (PMC12717851; doi:10.7717/peerj.20441)
Supplement: Supplemental Information 3 [file peerj-13-20441-s003.docx]

Supplementary Table 2. The correlation analysis between differential immune cells and biomarkers

|  | gene | cell | Correlation | Pvalue |
| --- | --- | --- | --- | --- |
| 1 | ACSL1 | Macrophage M0 | 0.488821 | 2.53E-10 |
| 2 | TSPO | Macrophage M0 | 0.503824 | 5.76E-11 |
| 3 | ALAS1 | Macrophage M0 | 0.334592 | 3.04E-05 |
| 4 | ALDH5A1 | Macrophage M0 | -0.0814 | 0.323679 |
| 5 | MTHFD2 | Macrophage M0 | -0.03732 | 0.65136 |
| 6 | PDSS1 | Macrophage M0 | 0.144098 | 0.079553 |
| 7 | ACSL1 | NK cell activated | -0.49383 | 1.56E-10 |
| 8 | TSPO | NK cell activated | -0.42842 | 5.02E-08 |
| 9 | ALAS1 | NK cell activated | -0.37748 | 2.08E-06 |
| 10 | ALDH5A1 | NK cell activated | 0.22164 | 0.006597 |
| 11 | MTHFD2 | NK cell activated | -0.30108 | 0.000191 |
| 12 | PDSS1 | NK cell activated | -0.38277 | 1.45E-06 |
| 13 | ACSL1 | Neutrophil | 0.84542 | 7.08E-42 |
| 14 | TSPO | Neutrophil | 0.635701 | 3.05E-18 |
| 15 | ALAS1 | Neutrophil | 0.577403 | 1.27E-14 |
| 16 | ALDH5A1 | Neutrophil | -0.26134 | 0.001285 |
| 17 | MTHFD2 | Neutrophil | 0.367056 | 4.13E-06 |
| 18 | PDSS1 | Neutrophil | 0.578636 | 1.09E-14 |
| 19 | ACSL1 | T cell CD4+ memory resting | -0.58453 | 5.02E-15 |
| 20 | TSPO | T cell CD4+ memory resting | -0.63173 | 5.70E-18 |
| 21 | ALAS1 | T cell CD4+ memory resting | -0.468 | 1.76E-09 |
| 22 | ALDH5A1 | T cell CD4+ memory resting | 0.192817 | 0.018475 |
| 23 | MTHFD2 | T cell CD4+ memory resting | -0.16954 | 0.038724 |
| 24 | PDSS1 | T cell CD4+ memory resting | -0.33772 | 2.53E-05 |
| 25 | ACSL1 | T cell CD4+ naive | -0.42142 | 8.69E-08 |
| 26 | TSPO | T cell CD4+ naive | -0.40551 | 2.89E-07 |
| 27 | ALAS1 | T cell CD4+ naive | -0.41646 | 1.27E-07 |
| 28 | ALDH5A1 | T cell CD4+ naive | 0.383846 | 1.35E-06 |
| 29 | MTHFD2 | T cell CD4+ naive | -0.46616 | 2.08E-09 |
| 30 | PDSS1 | T cell CD4+ naive | -0.39263 | 7.32E-07 |
| 31 | ACSL1 | T cell CD8+ | -0.75836 | 4.11E-29 |
| 32 | TSPO | T cell CD8+ | -0.59505 | 1.22E-15 |
| 33 | ALAS1 | T cell CD8+ | -0.64217 | 1.08E-18 |
| 34 | ALDH5A1 | T cell CD8+ | 0.412592 | 1.71E-07 |
| 35 | MTHFD2 | T cell CD8+ | -0.53783 | 1.52E-12 |
| 36 | PDSS1 | T cell CD8+ | -0.65631 | 1.03E-19 |
| 37 | ACSL1 | T cell regulatory (Tregs) | 0.466953 | 1.94E-09 |
| 38 | TSPO | T cell regulatory (Tregs) | 0.54619 | 5.82E-13 |
| 39 | ALAS1 | T cell regulatory (Tregs) | 0.467522 | 1.84E-09 |
| 40 | ALDH5A1 | T cell regulatory (Tregs) | -0.17492 | 0.032874 |
| 41 | MTHFD2 | T cell regulatory (Tregs) | 0.129628 | 0.115109 |
| 42 | PDSS1 | T cell regulatory (Tregs) | 0.266396 | 0.001024 |
