# Supplementary material for: Bioinformatics-based identification and validation of mitochondria-related genes associated with neonatal sepsis
Source: PeerJ. 2025 Dec 17;13:e20441. doi: 10.7717/peerj.20441 (PMC12717851; doi:10.7717/peerj.20441)
Supplement: Supplemental Information 7 [file peerj-13-20441-s007.doc]

# QPCR Experiment Report

# 1 Reagents

| **PCR-related Reagents** | **Manufacturer** |
| --- | --- |
| Water-free Ethanol | Chengdu Kelong Chemical Products Co., Ltd. |
| TRizol | Ambion |
| Primer | Qingke Biology |
| chloroform | Chengdu Gurda Adhesive Industry Co., LTD |
| RNase-free water | Saiweier |
| Isopropyl alcohol | Chengdu Kelong Chemical Products Co., Ltd. |

# 2 Instruments

| **PCR -related Instruments** | **Manufacturer** |
| --- | --- |
| S1000™ Thermal Cycler Ordinary PCR instrument | BIO-RAD |
| CFX ConnectReal-time quantitative fluorescence PCR instrument | BIO-RAD |
| 96-well quantitative PCR plate | Saiweier |
| PCR plate sealing film | LABSELECT |
| Chemiluminescence imaging system ChemiScope6100 | Shanghai Qinxiang Scientific Instrument Co., LTD |
| H1 16KR Benchtop refrigerated high-speed centrifuge | Kecheng Instrument Company |
| SC-3610 Low-speed centrifuge | Anhui Zhongke Zhongjia Scientific Instrument Co., LTD |
| Vortex oscillator SCL-VS | SCLLOGEX |
| Electrophoresis apparatus（164-5050） | BIO-RAD |
| 10μl 、20μl、200ul、1000ul Pipette tip | Beijing Lanjieke Technology Co., LTD |
| Micropipette | thermo scientific |
| Grinding instrument | Saiweier |

# 3 Methods

## 3.1 Extraction of total RNA

For 5 pairs of frozen whole blood samples, samples 1-5 belong to the Control group, and samples 6-10 belong to the NESE group. For each sample, 600 µL of whole blood is mixed with 1 mL of TRIzol reagent and homogenized thoroughly. The mixture is then left on ice for 10 minutes to fully lyse the cells. Next, 300 µL of chloroform is added, and the mixture is shaken vigorously for 30 seconds. Afterward, it is left at room temperature for 10 minutes to allow the phases to separate. The sample is then centrifuged at 12,000 g and 4°C for 15 minutes, resulting in a clear separation into three phases. The RNA is retained in the upper aqueous phase, which is colorless.

Carefully transfer the upper aqueous phase into a new EP tube, taking care not to include the middle and lower phases (if accidental contamination occurs, gently expel the unwanted layers). Then, add an equal volume of ice-cold isopropanol, invert the tube to mix, and leave it at room temperature for 10 minutes. (For small sample volumes, the mixture may be stored at -20°C overnight to improve RNA yield.) Afterward, centrifuge at 12,000 g and 4°C for 10 minutes. A white RNA pellet should be visible at the bottom of the tube (for smaller sample volumes, the pellet may not be visible, which does not affect the procedure). Gently tilt the tube to discard the supernatant, being careful not to discard the pellet. Use absorbent paper to remove moisture from the tube's opening.

Add 1 mL of 75% ethanol to the pellet, invert the tube a few times to resuspend the pellet, and let it sit for 2 minutes. Then, centrifuge the sample at 7,500 g and 4°C for 5 minutes to re-pellet the RNA. Repeat this wash step twice. Discard the supernatant, and carefully invert the tube on absorbent paper. Use a 10 µL pipette tip to carefully remove any remaining liquid, taking care not to disturb the pellet. Allow the pellet to air dry for 20 minutes, or dry it under a laminar flow hood to evaporate the ethanol and water. The RNA pellet should be transparent, but avoid over-drying, as this may affect subsequent RNA resuspension.

Finally, add 20-50 µL of RNase-free water to the dried RNA pellet and let it sit for 15 minutes to ensure complete dissolution. Take 1 µL of the dissolved RNA for concentration measurement using a NanoDrop, and record the RNA purity and concentration to calculate the appropriate amount for the reverse transcription step. The remaining RNA can be immediately used for reverse transcription or stored at -80°C for future use.

**3.1.1 RNA detection**

RNA Concentration Measurement:

Take 1 µL of RNA and measure its concentration using the NanoPhotometer N50.

Concentration detection result:

| Sample Number | **Sample Name** | Group | Concentration (ng/uL) | A260/A280 | A260/A230 |
| --- | --- | --- | --- | --- | --- |
| 1 | 李瑞洪 | Control | 2502.600 | 1.850 | 1.961 |
| 2 | 王佳蓉之子 | Control | 1908.700 | 2.020 | 1.869 |
| 3 | 发权 | Control | 1225.900 | 1.872 | 2.082 |
| 4 | 陈香之子 | Control | 1582.200 | 1.941 | 1.600 |
| 5 | 苏多珍之子 | Control | 1307.700 | 1.954 | 2.040 |
| 6 | 叶冷 | NESE | 1763.800 | 1.669 | 1.148 |
| 7 | 何甜甜之子 | NESE | 2315.800 | 2.024 | 1.218 |
| 8 | 陈海然 | NESE | 1152.100 | 1.835 | 1.479 |
| 9 | 王金香 | NESE | 1107.900 | 1.821 | 1.662 |
| 10 | 张海霞之子 | NESE | 1347.300 | 1.868 | 1.635 |

## 3.2 Reverse Transcription

The reverse transcription was performed using the SweScript First Strand cDNA Synthesis Kit from Sewei'er Company. Specifically, the components of the reverse transcription kit were taken out, thawed at room temperature, briefly centrifuged, and placed on ice. On ice, the following reagents and solutions were added in the order listed below:

| **Component** | **Volume** |
| --- | --- |
| 5x Reaction Buffer | 4ul |
| Primer | 1ul |
| SweScript RT I Enzyme Mix | 1ul |
| Total RNA | 0.1ng-5ug |
| NucleNESEe-Free Water | Add to 20ul |

After brief centrifugation, reverse transcription was performed on a standard PCR machine under the following conditions:

| **Temperature** | **Time** |
| --- | --- |
| 25℃ | 5min |
| 50℃ | 15min |
| 85℃ | 5s |
| 4℃ | hold |

## 3.3 Machine-based Detection

First, dilute the cDNA obtained from the above reverse transcription product 5-20 times with ddH2O (RNase/DNase free). Then, perform the qPCR reaction according to the following reaction system:

| **Component** | **Volume** |
| --- | --- |
| cDNA | 3ul |
| 2xUniversal Blue SYBR Green qPCR MNESEter Mix | 5ul |
| Forward primer (10µM) | 1ul |
| Reverse primer (10µM) | 1ul |

During the sample loading procedure, each time a liquid is aspirated, check the pipette tip for air bubbles. After dispensing the liquid into the well, also check the pipette tip for any remaining liquid. If there is any residual liquid, it should be expelled into the corresponding well. The loading operation must be completed in one continuous motion, without performing any other experimental tasks or unrelated activities during the process, in order to ensure the consistency of the duplicate wells.

After brief centrifugation, perform 40 cycles of reaction on the CFX96 Real-Time Quantitative PCR System under the following conditions. Amplification curves and melting curves should be generated, and the Ct values should be read.

The amplification conditions are as follows:

|  | **Temperature** | **Time** |
| --- | --- | --- |
| Pre-denaturation | 95℃ | 1min |
| Denaturation | 95℃ | 20s |
| Annealing | 55℃ | 20s |
| Extension | 72℃ | 30s |

**The relevant primer sequences are as follows:**

| **primers** | **sequences** |
| --- | --- |
| MTHFD2 F | GAACTGGCATTCCAACCCTA |
| MTHFD2 R | CCCATCTGTGTGCAGTAACA |
| PDSS1 F | GACCAATTATTGTGGCGCTAATG |
| PDSS1 R | AGACTAGCAGTGTGGATCATTTC |
| TSPO F | CCTACCTGGTCTGGAAAGAG |
| TSPO R | TCGGGCACCAAAGAAGATG |
| ALDH5A1 F | TGTACTGTCGTGGTGAAGCC |
| ALDH5A1 R | ACACCTGAAGGAATCCCAGC |
| ALAS1 F | GGTTGTGTTGGAGGGTACAT |
| ALAS1 R | CAGAGAGGTGGTGAAGATGAAG |
| ACSL1 F | AAAGACAGATGGGAGGAGACC |
| ACSL1 R | GTTGGTCGGAAGAGTACGCA |
| GAPDH F | CGAAGGTGGAGTCAACGGATTT |
| GAPDH R | ATGGGTGGAATCATATTGGAAC |

## 3.4 Result Analysis

First, examine the melting curve. If the curve is a smooth single peak, it can be determined that the primer specificity is good. If the curve is not smooth but has only one large peak, it indicates that the primer specificity is moderate, and the data can still be used. If the curve shows two or more peaks, the primer specificity is poor, and the primers need to be redesigned for repeated experiments.

The relative expression of the gene is calculated using the 2^–△△Ct^ method, as follows:

Step 1: Calculate △Ct = Ct (target gene) - Ct (reference gene);

Step 2: Calculate △△Ct = △Ct (experimental group) - △Ct (control group);

Finally, calculate 2^-△△Ct^ value and perform statistical analysis using GraphPad Prism 5 to calculate the p-value.
