# Supplementary material for: Bioinformatics-based identification and validation of mitochondria-related genes associated with neonatal sepsis
Source: PeerJ. 2025 Dec 17;13:e20441. doi: 10.7717/peerj.20441 (PMC12717851; doi:10.7717/peerj.20441)
Supplement: Supplemental Information 8 [file peerj-13-20441-s008.docx]

| **Sample Number** | **Original (Non-English)** | **English Translation** |
| --- | --- | --- |
| 1 | 李瑞洪 | Ruihong Li |
| 2 | 王佳蓉之子 | Son of Jiarong Wang |
| 3 | 发权 | Quan Fa |
| 4 | 陈香之子 | Son of Xiang Chen |
| 5 | 苏多珍之子 | Son of Duozhen Su |
| 6 | 叶冷 | Leng Ye |
| 7 | 何甜甜之子 | Son of Tiantian He |
| 8 | 陈海然 | Hairan Chen |
| 9 | 王金香 | Jinxiang Wang |
| 10 | 张海霞之子 | Son of Haixia Zhang |
